# A time and motion study of subcutaneous versus intravenous trastuzumab in patients with HER2‐positive early breast cancer

**DOI:** 10.1002/cam4.573

**Published:** 2016-01-25

**Authors:** Erwin De Cock, Xavier Pivot, Nik Hauser, Sunil Verma, Persefoni Kritikou, Douglas Millar, Ann Knoop

**Affiliations:** ^1^ United BioSource CorporationBarcelonaSpain; ^2^ CHU Jean MinjozBesançonFrance; ^3^ Department of Gynecology and ObstetricsKantonsspital Baden AGBadenSwitzerland; ^4^ Sunnybrook Odette Cancer CentreTorontoOntarioCanada; ^5^ United BioSource CorporationHammersmithUnited Kingdom; ^6^ F. Hoffmann‐La Roche LtdBaselSwitzerland; ^7^ Copenhagen University HospitalCopenhagenDenmark

**Keywords:** Breast cancer, human epidermal growth factor receptor 2, subcutaneous, time factors, trastuzumab

## Abstract

Within PrefHer (NCT01401166), patients and healthcare professionals (HCPs) preferred subcutaneous (SC) over intravenous (IV) trastuzumab. We undertook a prospective, observational time and motion study to quantify patients’ time in infusion chairs and active HCP time in PrefHer. Patients with HER2‐positive early breast cancer received four adjuvant cycles of SC trastuzumab (600 mg fixed dose via SC single‐use injection device [SID, Cohort 1] or SC handheld syringe [HHS, Cohort 2]) then four cycles of standard IV trastuzumab or the reverse sequence. Generic case report forms for IV and SC management, both in the treatment room and the drug preparation area, were tailored to reflect center practices. Patient chair time and active HCP time were recorded. We compared pooled Cohort 1 + 2 IV with Cohort 1 SC SID and Cohort 2 SC HHS mean times across eight countries and individually within them utilizing a random intercept generalized linear mixed‐effects model. Per session, the SC SID saved a mean of 57 min of patient chair time versus IV (range across countries: 47–86; *P *<* *0.0001); the SC HHS saved 55 min (40–81; *P *<* *0.0001). Active HCP time was reduced by a mean of 13 min per session with the SC SID (range across countries: 4–16; *P *<* *0.0001) and 17 min with the SC HHS (5–28; *P *<* *0.0001) versus IV. SC trastuzumab, delivered via SID or HHS, saved patient chair and active HCP times versus IV infusion, supporting a transition to either SC method.

## Introduction

Breast cancer is the most common cancer in women 1, and trastuzumab (Herceptin^®^, F. Hoffmann‐La Roche Ltd, Basel, Switzerland)‐containing regimens are standard of care for HER2‐positive disease 2, 3, 4. Subcutaneous trastuzumab (Herceptin^®^ SC, F. Hoffmann‐La Roche Ltd), administered via handheld syringe (HHS), was approved in this indication by the European Medicines Agency following the HannaH study 5. An SC single‐use injection device (SID), which automatically injects SC trastuzumab into the thigh, is bioequivalent to the HHS 6. While intravenous (IV) trastuzumab is administered as a weight‐based dose using an initial 90‐min infusion followed by subsequent 30‐min infusions over 18 3‐weekly cycles 7, SC trastuzumab is administered as a fixed 600 mg dose over 2–5 min 7, which may result in reduced use of healthcare resources. However, comparisons between IV infusion and SC injection times are insufficient when attempting to quantify reductions in patient chair time and/or healthcare professional (HCP) time with SC. Indeed, HCPs typically manage multiple patients simultaneously and are sometimes not actively engaged during entire administrations. Also, activities performed in the treatment room pre‐ and post‐infusion/‐injection, as well as drug preparation and dispensing activities typically performed in a drug preparation area (DPA), need to be considered.

We conducted a time and motion (T&M) study to quantify patient chair time and active HCP time associated with SC and IV trastuzumab within the PrefHer trial (NCT01401166), where patients with HER2‐positive early breast cancer were given four cycles of SC trastuzumab (by SID [Cohort 1] or HHS [Cohort 2]) followed by four cycles of IV or vice versa as part of 18 standard cycles 8, 9. We report Cohort 1 + 2 IV versus Cohort 1 SID versus Cohort 2 HHS T&M data.

## Methods

### Study design

This was a multinational, multicenter, observational T&M study, performed as a substudy to PrefHer. Two types of time data were collected for IV, SC SID, and SC HHS processes: patient chair time and active HCP time (an HCP being defined as any personnel involved in SC and IV processes). Patient chair time (see study definitions in Table 1) included IV or SC trastuzumab administration time and was based on “time of day” measurements (h/min). Active HCP time was measured for chronologically listed, pre‐selected tasks (Table 2) for IV, SC SID, and SC HHS processes, both in the treatment room (time for administration) and DPA (time for preparation). In the DPA, only total time required for drug reconstitution (IV), SC SID dispensing, and SC HHS filling were measured. Interviews with nurses and pharmacy staff at each site, performed to tailor process flows, revealed that patient registration, blood sampling, and visits to the physician would be identical for IV and SC. Active HCP time was based on “stopwatch time” measurements (min/sec). Three generic case report forms (CRFs) were developed for data collection during outpatient consultations/day hospital visits (for each route of trastuzumab administration), conducted by trained observers who were not part of the facility care team. Data on a single patient's trastuzumab administration were recorded on each CRF, constituting one observation. Multiple observations could be performed for each patient.

**Table 1:** Study definitions

Term	Definition
Patient chair time	Time between entry and exit of infusion chair
Infusion duration	Time between initiation and completion of IV infusion
Active HCP time	Time actively dedicated by any staff member to pre‐specified tasks
Treatment room	The place where IV and SC treatments are being administered
Drug preparation area	The place where IV trastuzumab reconstitution, SC SID dispensing, and SC HHS filling before the actual injection takes place. Thus, “drug preparation area” can refer to the hospital pharmacy or to a special aseptic drug preparation area within the day oncology unit

HCP, healthcare professional; HHS, handheld syringe; IV, intravenous; SC, subcutaneous; SID, single‐use injection device.

**Table 2:**
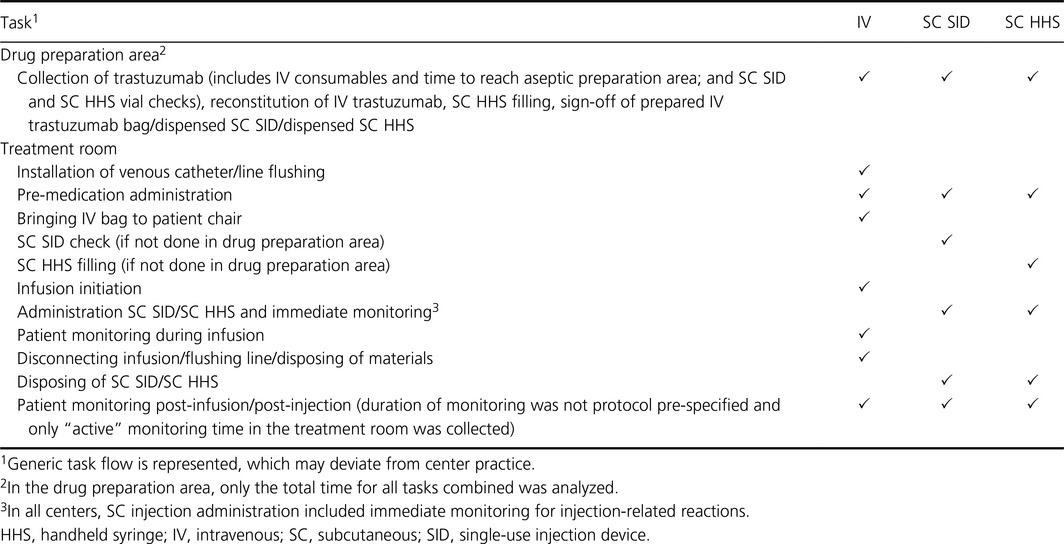
Chronological listing of observed tasks

T&M data collected per protocol were expected to be a good proxy for the real world because it was not expected that real‐world processes would deviate considerably from processes followed within PrefHer.

Patients provided written informed consent and were treated according to the PrefHer protocol. No exclusion criteria were applied to this study as it focused on HCPs. All centers agreed that staff could be observed. HCPs were not required to give separate consent.

### Data handling and statistical analyses

Data were analyzed using SAS 9.1 (SAS Institute Inc., Cary, NC).

This descriptive study was not powered to test formal hypotheses. Sample sizes for each route were dictated by observations performed within PrefHer. As the observations performed in the treatment room and DPA were independent, sample sizes in the two settings were expected to differ.

For incomplete CRFs, uncompleted tasks were marked as “0” if it was ascertained that tasks did not occur, and fields were left blank when it was unclear whether the task took place or not. Imputation (using the mean of available observations in that center) was performed if a task comprised ≥2 subtasks and if an absence of observed time for ≥1 subtasks would underestimate the total time. Logical tests were employed to identify erroneous data. Data that remained illogical post‐follow‐up with the providing site were treated as missing. Times were adjusted during quality control if unexpected events occurred, for example, if a task was performed by multiple HCPs, or if an adverse event was reported, in order to exclude any potential nonactive HCP time recorded due to the adverse event.

We applied two analytical approaches to estimate treatment room time: task‐based analysis (per country) and case‐based analysis (pooled countries). As part of the task‐based analysis, each task was treated as an independent data sample/variable, and was analyzed as such. To calculate the total active HCP time in the treatment room for each process, the mean times from each task were summed to a composite mean total time. As part of the case‐based analysis, each observation represented a case, and total active HCP time in the treatment room was calculated as the sum of all task times for a single observation. If a task time was missing, average time across all other cases in that center was imputed (otherwise, total active HCP time for that case would have been underestimated). For each process, the total case‐based time was then analyzed as a pooled variable across all countries.

For the DPA, a single composite time variable, “total drug preparation time,” was analyzed. IV infusion duration, SC SID and SC HHS injection time, and patient chair time data samples were also analyzed, and no imputation was needed for these.

For all variables, a random intercept generalized linear mixed‐effects model tested whether time was clustered by center. If a statistically significant center effect was detected (*α *= 0.05), adjusted mean time was used. If no effect was detected, standard regression employing best goodness of fit was considered appropriate (gamma distribution was used in most cases).

#### Covariate analyses

As time could be correlated with various factors, a set of variables were identified as potential predictors of patient chair time and active HCP time.

Analyses were performed on the pooled country data samples (due to increased sample sizes compared to each individual country, and hence increased ability to detect effects) using a random intercept generalized linear mixed‐effects model with “center” as the random effect and the covariate to be tested as the fixed effect.

Covariate analyses explored the potential impact of the variable “first versus subsequent infusion” on infusion duration and patient chair time, and of the variable “level of HCP's experience with administering SC via SID (never, 1–5, 5–10, 10+ injections performed)” on SC SID injection administration time and patient chair time.

No covariate analyses were performed for the SC HHS observations, as no potential confounders were identified.

#### Post hoc exploratory analyses

Post hoc analyses on the pooled country samples explored differences in patient chair time (including IV infusion duration) and active HCP time between IV and SC processes. All testing was two‐sided (*α *= 0.05).

#### Extrapolation into real‐world numbers

Patient chair time and active HCP time per session were extrapolated to one year of adjuvant trastuzumab (assuming 18 cycles). To obtain a more accurate estimate of extrapolated patient chair time, different time estimates for first and subsequent infusion, obtained from covariate analyses, were used.

For each process, the ten different HCP types involved in the various tasks were grouped into four categories: nursing staff, physicians, pharmacists, and pharmacy assistants. The distribution of active HCP time by HCP type for a single process was calculated for each country and extrapolated to one year of adjuvant trastuzumab treatment.

Similarly, the distribution of active HCP time by each individual task was calculated, with a view to identifying the tasks that were mainly responsible for the time differences observed between IV and SC administration. The country‐specific distributions were averaged across all countries and applied to the case‐based analyses’ results, in order to show an expected distribution of process workflows across the participating countries.

Results were further extrapolated to the estimated number of patients with HER2‐positive early breast cancer treated with trastuzumab in Germany, France, Italy, Spain, and the UK during 2013 (EU‐5 countries). These countries were selected to estimate the potential impact of a transition from IV to SC trastuzumab within the main European trastuzumab‐using countries, assuming that the estimates of patient chair time and active HCP time would hold true per country. The analysis was based on the number of assumed new early breast cancer cases and the average trastuzumab treatment rate (Roche data on file).

Savings in patient chair time and total active HCP time, when switching all patients from IV to SC, were computed, both for the total population of the EU‐5 countries and per ten million population, assuming 18 trastuzumab sessions per adjuvant treatment course.

## Results

### Observations

Patients were enrolled between October 2011 and December 2012 8, 9 and T&M data were collected between December 2011 and September 2013. Numbers of observations are shown in Table 3.

**Table 3:** Number of centers and completed observations for IV, SC SID, and SC HHS groups

Country	Centers, *n*	Observations, *n*					
		IV		SC SID		SC HHS	
		Drug preparation area	Treatment room	Drug preparation area	Treatment room	Drug preparation area	Treatment room
Canada	5	50	50	36	36	0	0
France	5	43	55	21	21	63	109
Switzerland	2	27	25	11	11	16	22
Denmark	2	33	30	22	20	10	18
Italy	4	68	65	0	0	65	68
Russia	5	125	121	95	95	99	99
Spain	3	89	90	74	73	65	65
Turkey	3	20	21	0	0	34	35

Canada participated in the SC SID cohort only; Italy and Turkey participated in the SC HHS cohort only.

Observations excluded from analyses – France: one for IV drug preparation area, five for SC HHS drug preparation area; Spain: one each for IV drug preparation area, IV treatment room, SC SID drug preparation area, SC SID treatment room, SC HHS drug preparation area, SC HHS treatment room.

No observations were performed in one center in Switzerland for IV in the SC HHS cohort, or in one center in France for IV and drug preparation area in the SC HHS cohort.

HHS, handheld syringe; IV, intravenous; SC, subcutaneous; SID, single‐use injection device.

### Patient chair time

**Figure 1:**
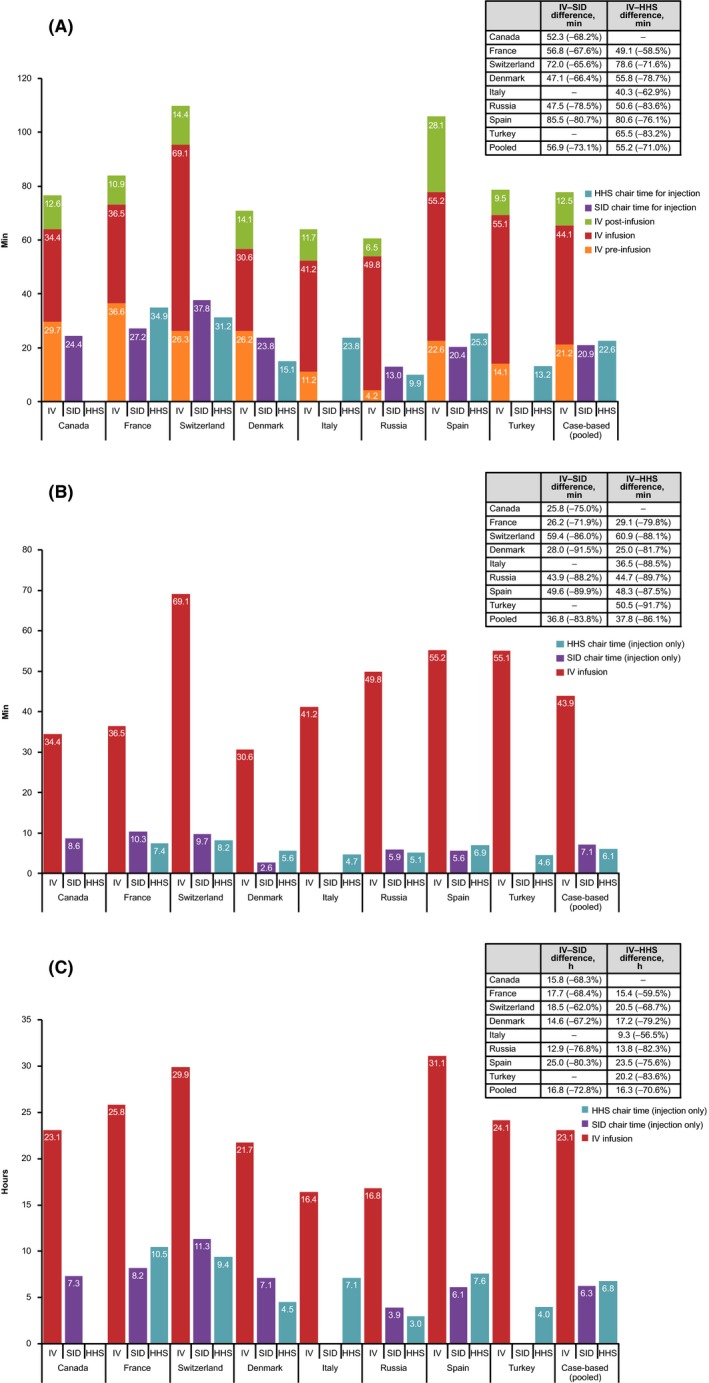
Patient chair time per IV, SC SID, and SC HHS administration by (A) infusion/injection stage per session by country and pooled, (B) infusion versus injection duration only per session by country and pooled, (C) infusion versus injection over 18 cycles by country and pooled (adjusted for first versus subsequent infusions). HHS, handheld syringe; IV, intravenous; SC, subcutaneous; SID, single‐use injection device.

Per session, the SC SID resulted in a mean reduction in patient chair time of 73.1% (20.9 versus 77.8 min with IV [*P *<* *0.0001]; range across countries: 47.1 to 85.5 min). Mean reduction with the SC HHS was 71.0% (22.6 min [*P *<* *0.0001]; range across countries: 40.3 to 80.6 min; Fig. 1A).

IV infusion, SC SID injection, and SC HHS injection time comparisons are shown in Fig. 1B.

Over 18 cycles, there was a mean reduction of 16.8 h with the SC SID (range across countries: 12.9 to 25.0 h) and 16.3 h with the SC HHS (range across countries: 9.3 to 23.5 h; Fig. 1C).

As expected, covariate analyses on the pooled dataset showed that subsequent IV infusions were associated with shortened infusion duration (42.0 versus 64.8 min, *P *<* *0.0001) and consequently with shortened patient chair time compared with that required for first IV infusions (75.3 versus 105.7 min, *P *<* *0.0001) (country‐specific data not shown due to small sample sizes and covariate imbalances between centers).

When extrapolating to the EU‐5 population, estimated patient chair time‐savings were 64,383 8‐h days with the SC SID (95% CI, 55,510–73,255), and 62,430 8‐h days (95% CI, 53,148–71,712) with the SC HHS (Table 4).

**Table 4:** Patient chair time and active HCP time using pooled results across all countries (treatment course of 18 sessions)

	IV		SC SID		SC HHS	
	Estimate	Likelihood ratio 95% confidence limits	Estimate	Likelihood ratio 95% confidence limits	Estimate	Likelihood ratio 95% confidence limits
Patient chair time						
Patient time in bed/chair, min	First: 105.69 Subsequent: 75.25	First: 92.42–118.96 Subsequent: 64.37–86.14	20.90	15.90–25.90	22.60	16.20–29.10
Per treatment course, h	23.08	19.78–26.39	6.27	4.77–7.77	6.78	4.86–8.73
Difference, h	–	–	16.81	14.30–19.33	16.30	13.97–18.63
EU‐5, 8‐h days^a^	88,393	75,737–101,049	24,010	18,266–29,751	25,963	18,611–33,430
Difference, 8‐h days	–	–	64,383	55,510–73,255	62,430	53,148–71,412
EU‐5 per 10,000,000 (8‐h days)	2,778	2,380–3,175	754	574–935	816	585–1,050
Difference, 8‐h days^a^	–	–	2,023	1,744–2,302	1,962	1,670–2,253
Active HCP time						
Total time in treatment room, min	17.90	14.07–21.78	11.20	9.05–13.43	9.80	8.46–11.09
Total time in drug preparation area, min	13.90	11.04–16.80	7.60	5.14–10.15	5.00	3.33–6.58
Total time, min	31.80	27.20–36.40	18.80	15.70–21.90	14.80	12.80–16.80
Per treatment course, h	9.54	8.16–10.92	5.64	4.71–6.57	4.44	3.84–5.04
Difference, h	–	–	3.90	2.24–5.56	5.10	3.60–6.60
EU‐5, 8‐h days	36,532	31,248–41,817	21,598	18,036–25,159	17,002	14,705–19,300
Difference, 8‐h days^a^	–	–	14,935	8,580–21,289	19,530	13,774–25,286
EU‐5 per 10,000,000 (8‐h days)	1,148	982–1,314	679	567–791	534	462–606
Difference, 8‐h days^a^	–	–	469	270–669	614	433–795

a IV versus SC SID or SC HHS.

EU‐5, France, Germany, Italy, Spain, UK; HCP, healthcare professional; HHS, handheld syringe; IV, intravenous; SC, subcutaneous; SID, single‐use injection device.

### Active HCP time

**Figure 2:**
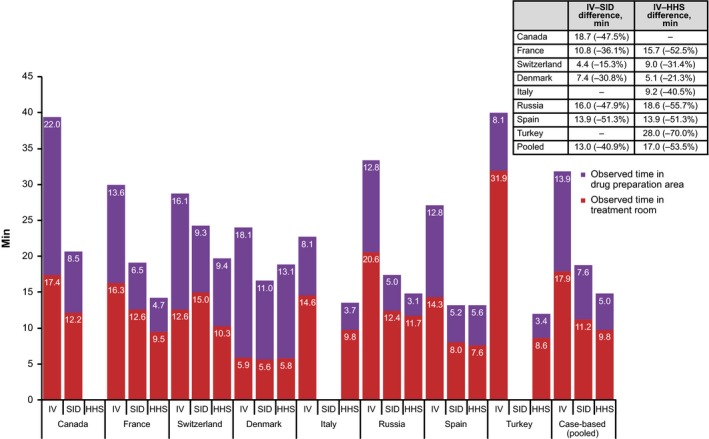
Active HCP time in the treatment room and drug preparation area per session by country and pooled. HCP, healthcare professional; HHS, handheld syringe; IV, intravenous; SID, single‐use injection device.

For a single administration, pooled country treatment room and DPA reductions were 40.9% with the SC SID and 53.5% with the SC HHS versus IV (Fig. 2, Table 4). Across countries, mean reductions ranged from 4.4 to 18.7 min with the SC SID, and from 5.1 to 28.0 min with the SC HHS (Fig. 2). Over 18 cycles, mean reductions from IV to SC ranged from 1.3 to 5.5 h with the SC SID, and from 1.6 to 8.4 h with the SC HHS across countries.

Nurses played the largest role overall, a fact which resulted in reductions to less than half of the IV administration time for both routes of SC administration (Table 5).

**Table 5:** Time‐savings by HCP type using pooled results across all countries

	Average time, min			Time‐savings	
	IV	SC SID	SC HHS	IV–SC SID	IV–SC HHS
Nursing staff	18.83	10.73	9.20	63%	56%
Physicians	2.51	2.23	1.26	2%	7%
Pharmacists	4.02	3.59	2.74	3%	8%
Pharmacy assistants	6.45	2.33	1.54	32%	29%

HCP, healthcare professional; HHS, handheld syringe; IV, intravenous; SC, subcutaneous; SID, single‐use injection device.

**Figure 3:**
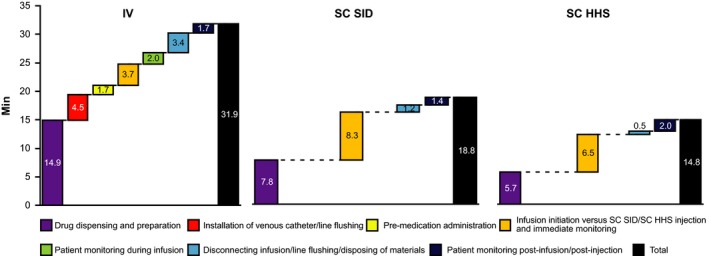
Active HCP time by task per session using pooled results across all countries. SC SID and SC HHS pre‐medication administration time = 0.1 min/1%. HCP, healthcare professional; HHS, handheld syringe; IV, intravenous; SC, subcutaneous; SID, single‐use injection device.

Applying an average proportion distribution by task to the mean pooled time (from the case‐based analysis) provided an indication of the process distribution by task across all countries (Fig. 3). Main drivers of active HCP IV time were “drug dispensing and preparation” (including reconstitution; accounting for approximately 50% of total observed time), “installation of venous catheter/line flushing,” and “disconnecting infusion/flushing line/disposing of materials,” which together accounted for approximately 25% of the total time. For SC, “drug dispensing and preparation” contributed approximately 40% to the total time for each method, and “administration SC SID/SC HHS and immediate monitoring” accounted for 44%. SC time‐savings were typically due to fewer DPA activities, and no installation/disconnection of peripheral catheters (or no permanent line flushing). However, savings were partially offset by increased SC injection time (compared with infusion initiation). It should be noted that, as the sample sizes differed by route of administration, comparisons of time to complete each session should be performed with caution.

Covariate analyses on the pooled dataset showed significant effects of SID experience on SC injection time (*P *=* *0.0006), with mean injection times of 8.3 min for HCPs with no experience, 7.5 min for HCPs having administered one to five injections, 6.7 min for HCPs having administered five to ten injections, and 6.4 min for HCPs having administered more than ten injections (country‐specific data not shown due to small sample sizes and covariate imbalances between centers).

Extrapolating to the EU‐5 population, estimated savings in active HCP time were 14,935 8‐h days for the SC SID (95% CI, 8,580–21,289), and 19,530 8‐h days (95% CI, 13,774–25,286) for the SC HHS (Table 4).

## Discussion

To our knowledge, this is the first T&M study to be run alongside a clinical trial, and it provides quantitative evidence to support previously published patient‐ and HCP‐reported preferences for SC trastuzumab 8, 9, 10. We demonstrated important reductions in patient chair time and active HCP with both SC methods across countries, trends which were confirmed statistically in pooled analyses. Together with the strong patient preference for SC trastuzumab regardless of delivery method 8, 9, 10 and the bioequivalence of the two 6, our data support the benefits of transitioning to either SC HHS or SC SID (if the SC SID becomes available).

Pooled data showed that reductions in patient chair time were driven by a reduction in trastuzumab administration time, and that at least 16 h of chair time could be freed up for a single patient treated with SC instead of IV over one year of adjuvant treatment (assuming 18 cycles). The number of hours of chair time freed up is a measure of center efficiency, whereby the same number of breast cancer patients is treated with fewer resources. This would lead to increased patient throughput, that is, an increased number of available appointments within day oncology units that operate at full capacity, thereby cutting waiting lists. From a funding perspective, this would result in alternative revenue for centers which have a fee‐for‐service or prospective payment structure. At the same time, a transition from IV to either of the SC routes would result in significant time‐savings for the patients themselves. Indeed, time‐saving was reported as one of the patients’ main reasons for preferring SC in PrefHer 8, 9, 10.

The covariate analyses (across all sites) showed a clear pattern of a reduction in infusion duration, and consequently patient chair time, for subsequent versus first infusions (as could reasonably be expected). Reductions in SC SID injection time also reflect increasing SC SID proficiency across subsequent infusions. Therefore, SC SID process time likely represents a conservative estimate, and further active time reductions could be expected with future real‐world application.

Limitations of the current analyses include those imposed by the running of the study alongside PrefHer: the centers and potential numbers of observations were directly dictated by the parent trial. Given the imbalances in the sample sizes and sample composition between IV and SC, the results of the pooled analysis need to be interpreted with caution.

While the data were collected within the confines of a clinical study, centers were free to prepare and administer trastuzumab as they would in clinical practice; thus, the data are expected to provide a reasonable approximation of real‐world practice.

By design, T&M studies focus on “dynamic” processes, resulting in time endpoints that are prone to variability. In the absence of prior information, it was not possible to define predictors of process flows and potential confounders of time. Indeed, a high level of heterogeneity was observed in terms of the task decomposition of some activities for all routes of administration, both among countries, and among centers. Variability in task composition and time (Fig. 3) may have been due to differences in individual center practices and different staff performing and measuring activities. For example, time for “installation of venous catheter/line flushing” depends on the proportion of patients requiring a peripheral catheter rather than a previously installed permanent line. In Russia, this task was combined with “infusion initiation.” In all centers, SC administration includes immediate monitoring for injection‐related reactions. The SC SID check (a specific procedure whereby a button is pressed to check proper functioning) could be performed in the DPA (Spain and Russia) or in the treatment room (other countries). For the SC HHS, “bringing trastuzumab to patient bed/chair” could constitute a separate step (France, Switzerland, and Italy), or could be combined with “administration of SC SID/SC HHS and immediate monitoring” (other countries). The HHS could be filled in the DPA (Switzerland), the treatment room (Spain, Denmark, and Turkey), or in either setting (Italy, Russia, and France). Therefore, results are not generalizable to the whole of each of the specific countries involved, or worldwide; however, the data provide a basis for expectations in a real‐world setting, and our case‐based analyses provide the most robust evidence for differences in time between IV and SC. Although we compared results for IV, SC SID, and SC HHS, this was a descriptive study, and the design was dictated by the clinical trial. To overcome any limitations resulting from the descriptive nature of the study, we designed this study to be as comprehensive as possible, including: a clear concept of active time, resulting in conservative estimates of time‐savings (indeed, it may be argued that some “non‐active” time is also attributable to the IV or SC processes); a focus on accurate process‐mapping to identify trastuzumab‐related tasks that were expected to differ between IV and SC; center initiation interviews to adjust generic CRFs to reflect center practices while maintaining core process flow within a country, allowing pooling of data; and thorough observer training and data management/quality assurance processes to limit measurement‐related variability and increase the overall quality of the data.

In conclusion, this study showed that, across a selection of countries and centers, a transition from IV to SC trastuzumab, regardless of SID or HHS administration, led to substantial reductions in patient chair time and active HCP time. Shorter patient chair time would reduce the amount of time that patients spend in hospitals, could reduce waiting lists, and could increase center capacity and throughput. The HCP time‐savings could allow more time to be dedicated to other patient care activities, and therefore increase the overall staff efficiency within treatment centers.

## Conflict of interest

EDC: Employee of United BioSource Corporation and has carried out this research on behalf of F. Hoffmann‐La Roche Ltd. XP: Consultant with honoraria: F. Hoffmann‐La Roche Ltd, TEVA, Amgen, Pierre Fabre, Eisai, Novartis, GSK. NH: None. SV: Advisory board: F. Hoffmann‐La Roche Ltd. PK: Employee of United BioSource Corporation and has carried out this research on behalf of F. Hoffmann‐La Roche Ltd. DM: Employee of F. Hoffmann‐La Roche Ltd. AK: Honoraria: Roche. Consulting/advisory roles: Roche, Pierre Fabre. Travel, accommodations, expenses: Roche, Novartis.
